# A new score for airway assessment using clinical and ultrasound parameters

**DOI:** 10.3389/fmed.2024.1334595

**Published:** 2024-02-14

**Authors:** Nekari De Luis-Cabezón, Diana Ly-Liu, Pablo Renedo-Corcostegui, Francisco Santaolalla-Montoya, Aitor Zabala-Lopez de Maturana, Jose Carlos Herrero-Herrero, Eugenio Martínez-Hurtado, Raúl De Frutos-Parra, Amaia Bilbao-Gonzalez, Miguel Angel Fernandez-Vaquero

**Affiliations:** ^1^Department of Anesthesiology, Osakidetza Basque Health Service, Basurto University Hospital, Bilbao, Spain; ^2^Instituto IIS Biobizkaia, Barakaldo, Spain; ^3^Department of Otorhinolaryngology, Osakidetza Basque Health Service, Basurto University Hospital, Bilbao, Spain; ^4^Department of Anaesthesiology, Hospital Universitario Infanta Leonor, Madrid, Spain; ^5^Unidad de Investigación e Innovación, RICAPPS, Osakidetza Basque Health Service, Basurto University Hospital, Bilbao, Spain; ^6^Department of Anaesthesiology, University Clinic of Navarra, Madrid, Spain

**Keywords:** anesthesia, airway management, intubation, difficult laryngoscopy, ultrasonography, scores

## Abstract

**Background:**

Over the last few years, ultrasonography has been introduced as the fifth pillar to patient’s bedside physical examination. Clinical assessments aim to screen and look for airway difficulties to predict difficult intubations, but none have demonstrated a significant predictive capacity. Recent systematic reviews have established a correlation between ultrasound imaging and difficult direct laryngoscopy. The primary objective of this study was to determine whether the utilization of ultrasonography to examine the upper airway could accurately predict difficult direct laryngoscopy.

**Methods:**

This is a prospective observational study including 102 adult patients that required general anesthesia for elective surgery. Preoperatively, clinical airway assessments were performed. Data such as Mallampati-Samsoon grade (MS), upper lip bite test (ULBT), thyromental (TMD) and sternomental distance (SMD), cervical circumference (CC) and the Arné risk index were collected. Ultrasound evaluation was taken at five different levels in two planes, parasagittal and transverse. Therefore, the following measurements were registered: distance from skin to hyoid bone (DSHB), distance from skin to thyrohyoid membrane (DSTHM), distance from skin to epiglottis (DSE), distance from skin to thyroid cartilage (DSTC) and distance from hyoid bone and thyroid cartilage (DHBTC). Patients were divided into two groups based on the difficulty to perform direct laryngoscopy, according to Cormack-Lehane (C-L) classification. Grades I and II were classified as easy laryngoscopy and grades III or IV as difficult. Logistic regression models and the Receiver Operating Characteristic (ROC) curve was employed to determine the diagnostic precision of ultrasound measurements to distinguish difficult laryngoscopy (DL).

**Results:**

The following risk score for DL was obtained, DSTHM ≥ 1.60 cm (2 points), DSTC ≥ 0.78 cm (3 points) and gender (2 points for males). The score can range from 0 to 7 points, and showed and AUC (95% CI) of 0.84 (0.74–0.95). A score of 5 points or higher indicates a 34-fold increase in the risk of finding DL (*p* = 0.0010), sensitivity of 91.67, specificity of 75.56, positive predictive value of 33.33, and negative predictive value of 98.55.

**Conclusion:**

The use of ultrasonography combined with classic clinical screening tests are useful tools to predict difficult direct laryngoscopy.

## Introduction

Management of difficult airway (DA) is one of the most challenging situations a physician can deal with in his clinical practice. Up to 30% of deaths related to anesthesia are due to the inability to maintain a permeable airway and provide adequate ventilation or a tracheal intubation. The optimal approach to mitigate these complications consists of an early identification of those patients with a higher risk of DA management. Unfortunately, this is an intricate endeavor, as evidenced by the multiple classifications published over the last years (1).

Morbidity and mortality prevalence due to DA during anesthesia induction varies from 1/10,000 to 1/100,000 patients. Almost two thirds of the complications related to airway management occurs during induction (2). Incidence of a “can’t intubate, can’t oxygenate” (CICO) scenario is 1/50,000 patients. Tracheal intubation failure occurs in 1/2,000 scheduled procedures, rising to 1/200 in emergency settings. Difficult intubation percentage is variable, as it ranges from 1.2 to 3.8% depending on the series (3). Difficult laryngoscopy (DL) implies a different concept, which is defined as the presence of grades III or IV based on the Cormack-Lehane classification using conventional laryngoscopy.

The evaluation and anticipation of a DA represent the first stage in the management of the airway. The negation to recognize a problematic airway may result in life-threatening complications, including brain injury and death (3). Multiple DA predictors have been described. Clinical detection methods include a detailed revision of the clinical history, comorbidities, previous anesthesia exposures, physical status assessment and conditions associated with DA management (1). Currently, the clinical tests commonly used to predict DA, as Mallampati-Samsoon grade (MS), upper lip bite test (ULBT), thyromental (TMD), sternomental distance (SMD), cervical circumference (CC) and neck mobility, exhibit unreliable predictive effects as well as limited sensitivity and specificity (4, 5).

Over the last few years, ultrasonography has been introduced as the fifth pillar to patient’s bedside physical examination: inspection, palpation, percussion, auscultation and insonation (6). For airway assessment and management, point-of-care ultrasound (PoCUS) has entered routine clinical practice answering open focused questions on diagnosis, narrowing differential diagnosis and guiding bedside procedures (7, 8). Several systematic reviews have correlated ultrasound measurements with DL (9–11).

The primary objective of this study is to predict DA considered as C-L III-IV by ultrasound parameters. The secondary objectives are to establish a DL risk score based on statistically significant variables using a combined model which includes ultrasound and clinical parameters; and to determine if this score would be superior for DL prediction than the clinical parameters used in routine clinical practice.

## Materials and methods

Ethical approval for this study (protocol N^°^ 43.17 CEIHUB) was provided by the Clinical Ethic Committee of Basurto University Hospital, Bilbao, Spain, on 20th September 2017. The study was conducted in accordance with the Helsinki Declaration, Good Clinical Practice guidelines, and the Spanish legislation for biomedical research.

This is a prospective, cross-sectional, single-center observational study which enrolled 102 patients scheduled for elective surgery undergoing general anesthesia between May 2015 and September 2017 at Basurto University Hospital. All scheduled patients were potential participants. Inclusion criteria were males and females aged 18 to 90 years old, American Society of Anesthesiologists (ASA) physical status classification I-III, scheduled for elective surgery undergoing general anesthesia with orotracheal intubation.

Exclusion criteria were pregnancy, cognitive impairment or incapacity to provide consent, pre-existing cervical disease or contraindications to forced cervical extension, patients with a known difficult airway, Body Mass Index (BMI) > 35 Kg.m^2^, allergy to ultrasound gel and patients who refused to participate in the study.

Patients who met inclusion criteria were previously selected the day before elective surgery based on the surgical schedule. Then, these patients were informed about the study protocol on the day of surgical intervention at the pre-surgical unit. Study objectives were explained in detail and written informed consent was delivered to each patient so they could read and understand all relevant aspects of the study. Questions could be asked and doubts were clarified before signing the consent. Thus, patient selection was carried out randomly because those patients who met the inclusion criteria and accepted to participate in the study were finally recruited. Only those who signed informed consent were finally included.

We designed the study performing an upper airway ultrasound exploration, procedure with no recognized secondary effects nor contraindications, using a portable ultrasound machine with a 5 to 14 MHz linear array transducer (Edge II, Sonosite^®^, USA). Tracheal intubation was performed using conventional laryngoscope with Macintosh blades.

At the pre-surgical area, demographic variables (age, gender, weight, body mass index and ASA physical status) were collected and several clinical airway tests were evaluated, including MS grade, ULBT, SMD, CC and the Arné risk index (ARI).

**FIGURE 1:**
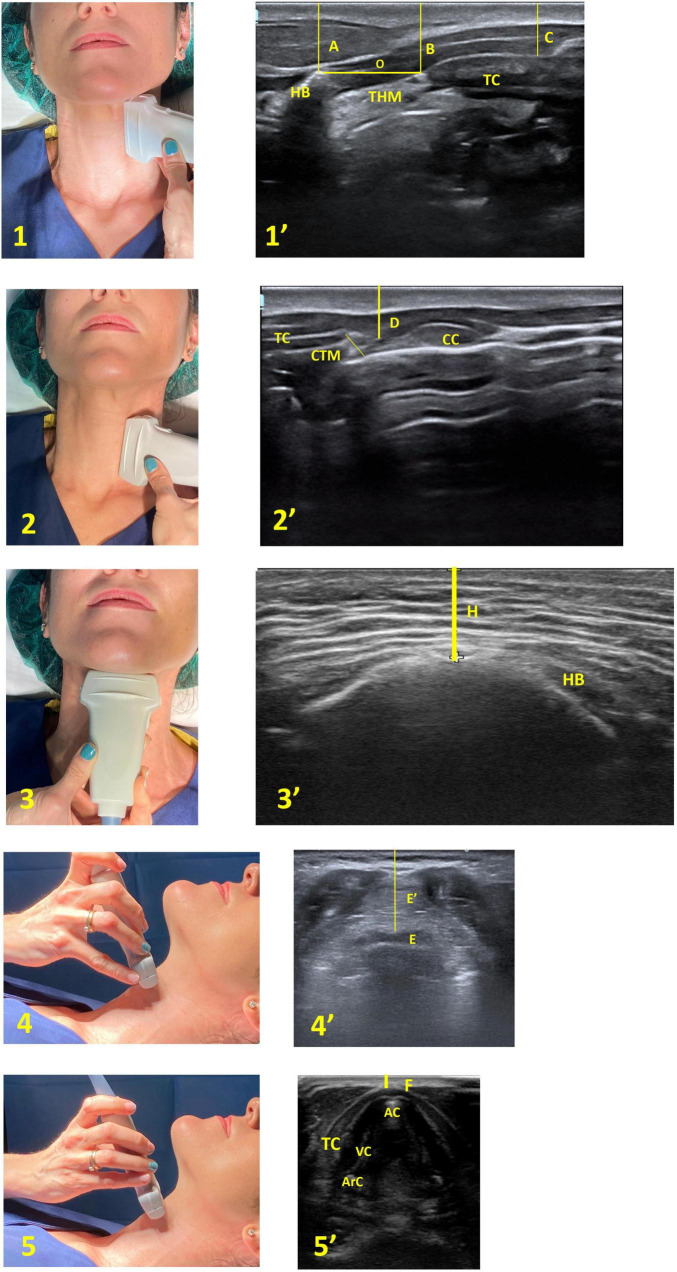
Ultrasound measurements of pre-tracheal tissues. Left panel different neck levels with ultrasound probe. (1) and (2) patient in sniffing position and probe in parasagittal plane. (3) (4) and (5) probe in transverse plane. (1): Thyrohyoid membrane level; (2): cricothyroid membrane level; (3): Hyoid bone level; (4): Epiglottis level; (5): Thyroid cartilage level. Right panel corresponding ultrasound images: (1’): (HB) Hyoid bone (THM) Thyrohyoid membrane (TC) Thyroid cartilage (A) Measure the distance from the hyoid bone to the skin (B) Distance from the thyrohyoid membrane to the skin (C) Distance from the thyroid cartilage to the skin (O) Distance from hyoid bone to thyroid cartilage. (2’): (TC) Thyroid cartilage (CTM) Cricothyroid membrane (CC) Cricoid cartilage (D) Measure the distance from the cricothyroid membrane to the skin. (3’): (HB) Hyoid bone (H’) Distance from Skin to Hyoid Bone. (4’): (E) Epiglottis (E’) Measure the distance from the epiglottis to the skin. (5’): (AC) Anterior commissure (TC) Thyroid cartilage (VC) Vocal cords (ArC) Arytenoid cartilage (F) Distance from skin to thyroid cartilage.

Ultrasound measurements were performed in the presurgical area by two experienced anaesthesiologists following the same examination protocol, with the patient in supine position and the head in the sniffing position. The ultrasound scanning technique was performed in the left parasagittal and transverse plane, from cranial to caudal. Five different levels were evaluated, distance from skin to hyoid bone (DSHB), distance from skin to thyrohyoid membrane (DSTHM), distance from skin to epiglottis (DSE), distance from skin to thyroid cartilage (DSTC) and distance from hyoid bone and thyroid cartilage (DHBTC) (Figure 1).

After completion of ultrasound assessments, standard non-invasive anesthesia monitoring (ECG, SpO2, non-invasive blood pressure, capnography, accelerometric neuromuscular monitoring and hypnotic depth) was applied before general anesthesia induction. Preoxygenation was confirmed by an ETO_2_ greater than 90%, and general anesthesia was induced with propofol (1.8–2.5 mgkg^–1^), remifentanil (0.1–0.15 μgkgh^–1^ and rocuronium (0.6–1 mgkg^–1^). When adequate hypnotic depth (BIS below 50, Covidien, Mansfield, USA) and neuromuscular relaxation (TOF = 0) were achieved, direct laryngoscopy with a Macintosh blade sizes 3 or 4 (Riester^®^, Jungigen, Alemania) was performed to evaluate the C-L grade and tracheal intubation was followed. Criteria to stop this procedure was SpO_2_ level below 91%. Before considering an unsuccessful direct laryngoscopy intubation, a maximum of two intubation attempts were allowed. In such cases, a videolaryngoscope (Airtraq^®^ Prodol Meditec, Vizcaya, Spain) was used as a rescue device.

Laryngoscopy vision was classified into two groups: easy (corresponding to C-L grades I and II) and difficult (for C-L grades III and IV). Previous studies have shown a 5 to 10% higher incidence of airway management complications with these higher C-L grades.

Due to the nature of ultrasound measurements performance and airway management, it was not possible to design a blinded study for the operators. However, the results of the ultrasound measurements were not disclosed to the anaesthesiologist who performed the laryngoscopy and intubation. Clinical and ultrasound evaluations were carried out by the same two investigators to minimize variability.

### Statistical analysis

Descriptive statistics included frequency tables for categorical variables and mean and standard deviation (SD) for continuous variables. Univariate analysis was used to see the individual association between sociodemographic, clinical and ultrasound variables with DL. The Chi-square test or Fisher’s exact test was used for the comparison of qualitative variables between easy and difficult laryngoscopy groups, and the Student’s *t*-test or Wilcoxon’s non-parametric test for quantitative variables. Univariate analyses were also performed by means of logistic regression models, and data was presented using odds ratio (OR) with 95% confidence interval (CI).

The continuous ultrasound variables were also considered as categorical. For the categorization, the Receiver Operating Characteristics (ROC) curve approach was used, considering as optimal cut-off value the one which maximized the sum of sensitivity and specificity. Then, we analysed the association of each categorized ultrasound measurements with DL by means of the logistics regression models and the area under the ROC curve (AUC). The accuracy, sensitivity, specificity, positive predictive value (PPV) and negative predictive value (NPV) of each indicator were also calculated.

Multivariate analysis was carried out using the logistic regression model. Variables with a significance of *p* < 0.15 were considered potential independent variables in the multivariate logistic regression model. In the final multivariate model, only those variables with *p* < 0.05 were maintained. Data was presented using OR with 95% CI. The predictive accuracy of the model was evaluated using the AUC for discrimination (12), and by comparing predicted and observed difficult laryngoscopy using the Hosmer and Lemeshow test for calibration (13).

A predictive risk score was developed from the final multivariate model. First, a weight was assigned to each predictor variable based on the estimated beta parameter of the multivariate model. By adding the weights of each predictor factor, the risk score was defined, in which a higher score indicated a greater risk of DL. The predictive accuracy of this risk score was determined through the AUC (14, 15) and the Hosmer-Lemeshow test (13). Next, the score was categorized into two groups (low risk and high risk) using the ROC curve method, considering the optimal cut-off point as the value that maximizes the sum of sensitivity and specificity. The performance of this categorization was studied by comparing the percentage of DL in each group, the AUC, and estimating the sensitivity, specificity, PPV and NPV.

A *p*-value < 0.05 was considered statistically significant. Data processing and statistical analyses were performed using SAS System for Windows, version 9.2 (SAS Institute Inc., Cary, NC, USA).

## Results

A total of 102 patients met the selection criteria for this study, of which 48 men (47.08%) and 54 women (52.94%), aged between 19 and 90 years old were included in the analysis. Direct laryngoscopy was classified as easy in 90 patients (88.24%) and difficult (C-L III-IV) in 12 cases (11.76%).

Twelve DL cases were detected, 9 were men (75%) and 3 were women (25%). No significant association was found between gender and C-L grade (*p* = 0.0508; Table 1). However, a trend is observed because its value is very close to be statistically significant with an OR (95% CI) of 3.92 (0.99–15.46). There was no association between age and C-L grade (*p* = 0.9656). Although half of the DL cases were obese, we could not detect a statistically significant relationship between obesity and C-L grade (*p* = 0.2981). A statistically significant association was found between CC and C-L grade, where the percentage of DL patients was much higher among patients with CC ≥ 43 cm than among those with CC ≥ 43 [35% vs. 6.10%, with an OR (95% CI) of 8.29 (2.28–30.10), *p* = 0.0013]. No significant association was found between C-L grade and MS (*p* = 0.2383), SMD (*p* = 0.8528), ULBT (*p* = 0.9492) and ARI (*p* = 0.1743) (Table 1).

**TABLE 1:** Demographic variables, clinical airway assessment and ultrasound measurements.

Variables	Easy laryngoscopy (*n* = 90)	Difficult laryngoscopy (*n* = 12)	OR (95% CI)	*p*-value
**Sociodemograthic:**				
**Gender (male/female) n (%)**				
male	39(81.25%)	9 (18.75%)	3.92 (0.99–15.46)	0.0508
female	51(94.44%)	3 (5.56%)	Ref.	
Age (years), mean ± SD	59.87 ± (15.36)	59.67 ± 14.23	0.99 (0.96–1.04)	0.9656
**BMI (kg/m^2^) n (%)**				
< 29.9	59(90.77%)	6(9.23%)	Ref.	
≥ 30	31(83.78%)	6(16.22%)	1.90 (0.57–6.40)	0.2981
**Clinical variables:**				
**MMS n (%)**				
I-II	79(89.77%)	9(10.23%)	Ref.	
III-IV	11(78.57%)	3(21.43%)	2.39 (0.56–10.22)	0.2383
**SMD (cm) n (%)**				
≤ 12.5	17(89.47%)	2(10.53%)	Ref.	
> 12.5	73(87.95%)	10(12.05%)	1.16 (0.23–5.81)	0.8528
**ULBT n (%)**				
I-II	82(88.19%)	11(11.83%)	Ref.	
III	8(88.89%)	1(11.11%)	0.93 (0.11–8.18)	0.9492
**CC (cm) n (%)**				
< 42	77(93.90%)	5(6.10%)	Ref.	
≥ 43	13(65.00%)	7(35.00%)	8.29 (2.28–30.10)	0.0013
**ARI n (%)**				
0-10	75(90.36%)	8(9.64%)	Ref.	
≥ 11	15(78.95%)	4(21.05%)	2.50 (0.67–9.38)	0.1743
**Ultrasound measurements: mean ± SD**				
DSHB *PS (cm)	1.10 ± 0.32	1.26 ± 0.39	4.06 (0.66–24.98)	0.1419
DSHB *T (cm)	0.86 ± 0.30	0.94 ± 0.32	2.27 (0.31–16.76)	0.4408
DSTHM *PS (cm)	1.17 ± 0.31	1.42 ± 0.40	9.43 (1.40–63.33)	0.037
DSTC *PS (cm)	0.82 ± 0.24	1.04 ± 0.35	20.83 (2.11–205.84)	0.0195
DSTC *T (cm)	0.37 ± 0.25	0.42 ± 0.23	1.95 (0.21–17.97)	0.4286
DHBTC *PS (cm)	1.29 ± 0.47	1.48 ± 0.40	2.27 (0.69–7.47)	0.0986
DSE *T (cm)	1.95 ± 0.30	2.33 ± 0.42	23.96 (3.52–163.17)	0.0013

BMI, body mass index; ASA, American Society of Anesthesiology; MMS, Modifed Mallampati score; SMD, sternomental distance; ULBT, upper lip bite test; CC, cervical circunference; ARI, Arné risk index; DSHB, distance from skin to hyoid; DSTHM, distance from thyrohyoid membrane; DSTC, distance from skin to thyroid cartilage; DHBTC, distance from hyoid bone to thyroid cartilage; DSE, distance from skin to epiglottis; *PS, parasagittal axis; *Transverse axis; OR, odds ratio; CI, confidence interval; SD, standard deviation. Ref. Reference group.

Regarding ultrasound continuous measurements, the mean airway distances were higher among patients with DL comparing with those with easy laryngoscopy (EL), although we only found statistically significant differences in the following measurements: DSTHM in parasagittal axis 1.17 ± 0.31 cm for EL vs 1.42 ± 0.40 cm for DL [OR (95% CI) = 9.43 (1.40–63.33), *p* = 0.0370], DSTC in parasagittal axis 0.82 ± 0.24 cm for EL vs. 1.04 ± 0.35 cm for DL [OR (95% CI) = 20.83 (2.11–205.84), *p* = 0.0195], and DSE in transverse axis 1.95 ± 0.30 cm for EL vs 2.33 ± 0.42 for DL [OR (95% CI) = 23.96 (3.52–163.17), *p* = 0.0013].

Table 2 shows the cut-off points of ultrasound measurements and the accuracy for predicting DL. Data obtained in parasagittal axis are described as follows. The DSHB with a cut-off point at 1.28 cm could be predicted with a PPV of 20.59% and a NPV of 92.65%. The DSTHM with a cut-off at 1.60 cm had a PPV of 37.50% and a NPV of 93.02%. The DSTC with a cut-off at 0.78 cm had a PPV of 19.30% and a NPV of 97.78%. The DHBTC with a cut-off at 1.23 cm had a PPV of 18.37% and a NPV of 94.34% (Table 2). On the other hand, regarding data obtained in transverse axis, the DSHB with a cut-off point at 0.65 cm could be predicted with a PPV of 14.29% and a NPV of 96.00%. The DSTC with a cut-off at 0.38 cm had a PPV of 15.91% and a NPV of 91.38%. The DSE with a cut-off of 2.10 cm had a PPV of 29.41% and a NPV of 97.06%. As previously, the ultrasound categorized measurements statistically associated with DL were parasagittal axis DSTHM ≥ 1.60 (*p* = 0.0018) with an AUC of 0.69, parasagittal axis DSTC ≥ 0.78 (*p* = 0.0272) with an AUC of 0.70, and transverse axis DSE (*p* = 0.0012) with an AUC of 0.78 (Table 2).

**TABLE 2:** Ultrasound measurements’ cut-off points and predictive accuracy.

US measurement	Cut-off point (cm)	OR (95% CI)	*p* value	AUC (95% CI)	Sensitivity	Specificity	PPV	NPV	Younden index
DSHB *PS	1.28	3.27 (0.95–11.21)	0.0599	0.64 (0.49–0.80)	58.33	70	20.59	92.65	0.28
DSTHM *PS	1.6	8 (2.16–29.61)	0.0018	0.69 (0.54–0.85)	50	88.89	37.5	93.02	0.39
DSTC *PS	0.78	10.52 (1.30–84.87)	0.0272	0.70 (0.61–0.80)	91.67	48.89	19.3	97.78	0.41
DHBTC *PS	1.23	3.75 (0.95–14.77)	0.0589	0.65 (0.52–0.79)	75	55.56	18.37	94.34	0.31
DSHB *T	0.65	4 (0.49–32.66)	0.1957	0.59 (0.50–0.69)	91.67	26.67	14.29	96	0.18
DSTC *T	0.38	2.01 (0.59–6.81)	0.2644	0.59 (0.43–0.74)	58.33	58.89	15.91	91.38	0.17
DSE *T	2.1	13.75 (2.81–67.32)	0.0012	0.78 (0.66–0.90)	83.33	73.33	29.41	97.06	0.57

AUC, Area Under ROC Curve; DSHB, distance from skin to hyoid bone; DSTHM, distance from skin to thyrohyoid membrane; DSTC, distance from skin to thyroid cartilage; DHBTC, distance from hyoid bone to thyroid cartilage; DSE, distance from skin to epiglottis; *PS, parasagittal axis; *Transverse axis; PPV, positive predictive value; NPV, negative predictive value; OR, odds ratio; CI, confidence interval.

**FIGURE 2:**
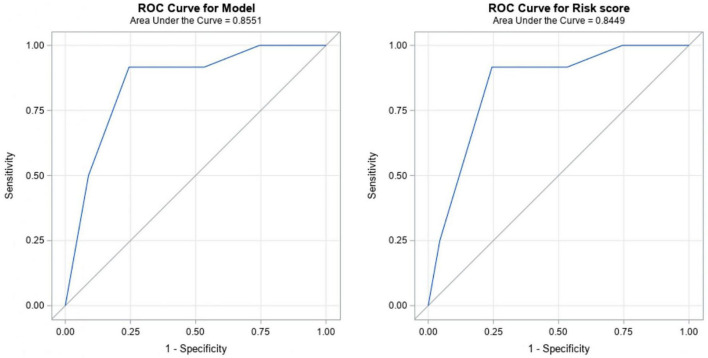
The Receiver Operating Characteristics (ROC) curve for the multivariate model (left) and for the derived difficult laryngoscopy risk score (right).

Regarding multivariate logistics regression analysis, a risk score for DL was obtained (Table 3). DSTHM in parasagittal plane ≥ 1.60 cm, DSTC in parasagittal plane ≥ 0.78 cm and male gender were independently associated with an increased risk of presenting a C-L grade ≥ III. Specifically, among those patients with the DSTHM in parasagittal plane ≥ 1.60 cm, the probability of a C-L grade ≥ III was 5.35 times higher (*p* = 0.0208); patients with a DSTC in parasagittal plane ≥ 0.78 cm, showed 8.94 times higher risk of DL (*p* = 0.0477); and men had 4.84 times the risk than women to present C-L grade ≥ III (*p* = 0.0390). This model showed a good discriminatory ability (AUC = 0.86) and an adequate calibration (Hosmer and Lemeshow, *p* = 0.6575) as shown in Table 3 and Figure 2.

**TABLE 3:** Multivariate logistic regression model for predicting difficult laryngoscopy and the performance of the risk score.

Variables	OR (95% CI)	*p*-value	Beta	Weight
**Multivariate model**				
DSTHM *PS				
< 1.60 cm	Ref.			0
≥ 1.60 cm	5.35 (1.29–22.16)	0.0208	1.6769	2
DSTC *PS				
< 0.78 cm	Ref.			0
≥ 0.78 cm	8.94 (1.02–78.23)	0.0477	2.191	3
**GENDER**				
Female	Ref.			0
Male	4.84 (1.08–21.65)	0.039	1.5775	2
AUC	0.86 (0.75–0.96)			
Hosmer-Lemeshow, *p*-value	0.6575			
**Risk score**	**OR** **(95% CI)**	***p*-value**	**Range**	
Score	2.20 (1.41–3.42)	0.0005	0–7	
AUC (95% CI)	0.84 (0.74–0.95)			
Hosmer-Lemeshow, *p*-value	0.4028			

OR, Odds Ratio; CI, Confidence Interval; Ref. Reference group; AUC, Area under the curve; DSTHM, distance from skin to thyrohyoid membrane; DSTC, distance from skin to thyroid cartilage; *PS, parasagittal axis.

Considering the multivariate model, a predictive tool for DL was derived (Table 3). Based on the beta parameters of the model, the weights were assigned as follows: 2 points if DSTHM ≥ 1.60 cm; 3 points if DSTC ≥ 0.78 cm, and 2 points if the patient is male. As a result, a risk score ranging from 0 to 7 points was derived (Table 3). The predictive accuracy of the risk score was satisfactory, with and OR (95% CI) of 2.20 (1.41–3.42) and statistically significant (*p* = 0.0005), good discriminatory ability (AUC = 0.84) and adequate calibration (Hosmer-Lemeshow, *p* = 0.4028) (Figure 2 and Table 3).

The cut-off point for this risk score is 5 points. Therefore, if the final score is 5 or higher, the likelihood of DL would be 34 times higher (*p* = 0.0010). This score has good ability of discrimination (AUC = 0.84), and has a 91.67% sensitivity and a 75.56% specificity. PPV is 33.33% and NPV 98.55% (Table 4).

**TABLE 4:** Predictive accuracy of the categorized risk score for predicting difficult laryngoscopy.

Risk score	n	Difficult laryngoscopy n (%)	OR (95% CI)	*p*-value	Sensitivity	Specificity	PPV	NPV	Younden index
**Score**									
<5	69	1 (1.45)	Ref.		91.67	75.56	33.33	98.55	0.67
≥5	33	11 (33.33)	34 (4.15–278.42)	0.001					
**AUC (95% CI)**			0.84 (0.74–0.93)						

OR, odds ratio; CI, confidence interval; AUC, area under the ROC curve; PPV, positive predictive value; NPV, negative predictive value.

## Discussion

Currently there is no single predictor that can accurately predict a difficult airway by itself. Thus, in this prospective and observational study, difficult airway clinical predictors and ultrasound measurements are compared and integrated to create a risk score to predict DL (16).

Within the ultrasound measurements to predict DL defined as C-L ≥ III using direct laryngoscopy with a Macintosh blade, a statistically significant association was found for the following distances: distance from skin to epiglottis in transverse axis, distance from skin to thyroid cartilage and distance from skin to thyrohyoid membrane in parasagittal axis.

In the present study, laryngeal measurements were taken in both transverse and parasagittal planes. Parasagittal plane measurements were performed instead of sagittal plane because in men, the laryngeal prominence has a 90-degree angle, while in women, the angle is approximately 120 degrees. Due to this increased angulation of the thyroid cartilage, the sagittal plane probe does not properly coapt with the skin and sometimes hinders the correct visualization of laryngeal structures. Therefore, to standardize and homogenize the examinations of all patients included in the study, in addition to what has been described in the literature, data collection was performed in the parasagittal plane. Furthermore, examination was performed on the left side due to the preference of right-handed examiners. No limitations were found in the parasagittal approach for locating laryngeal structures and for performing laryngeal measurements (17, 18).

Among all the ultrasound parameters studied, distance from skin to epiglottis with a cut-off point of 2.10 cm, showed the best predictive ability with a sensitivity of 83.33% and a specificity of 73.33%, for predicting difficulty in airway management in routine clinical practice. This result is similar to various studies and metanalysis reported in the literature (11, 17, 19–26). A higher cut-off point of 2.70 cm can be found in other studies (27). We believe that these variations could be due to the differences between the population samples studied. Furthermore, methodological heterogeneity in ultrasound measurements and laryngoscopy technique could cause bias.

The systematic review a meta-analysis published by Carsetti et al. (10) demonstrates that distance from skin to epiglottis is the most studied index test in literature to predict difficult direct laryngoscopy. It may help to rule out the probability of a true DL in a selected population with uncertain difficult airway based on clinical assessment (18).

In our study, the distance from skin to hyoid bone data differ depending on the planes studied. In transverse plane with a cut-off point of 0.65 cm, we did not find a statistically significant relationship (*p* = 0.1957) with DL. This result agrees with other studies, but curiously with those that have a small sample size (25, 28), so this could be one of the main reasons why the result is not significant. However, other studies that do find association with DL present higher cut-off points, most of them between 0.9 and 1.4 cm (11, 21, 23, 24, 29).

It is of special interest that in the parasagittal plane, a strong trend in distance from skin to hyoid bone was found, with a cut-off point of 1.28 cm, with a sensitivity of 58% and specificity of 70%, although not significant (*p* = 0.0599). This parameter has not been studied to our knowledge, so further research is needed to corroborate this data.

According to our results, DL is associated with distance from skin to thyrohyoid membrane in parasagittal plane, with a cut-off point of ≥ 1.60 cm (*p* = 0.0018). We have not found studies where this measurement was taken in order to compare the results obtained, since in all the publications, measurements were taken in the transverse plane. However, it is remarkable to highlight the importance of this measurement according to our results.

In the present work, distance from skin to thyroid cartilage ≥ 0.78 cm has been determined to increase the risk of DL in more than 10 times (*p* = 0.0272). It should be noticed that when a wide CI is obtained, the precision of the OR estimation is low, and increasing the sample size would improve this data. This result is supported by other studies (23). However, when we compare the results with samples of very diverse patients, as in the case of Ezri (30) carried out in 50 morbidly obese patients, the cut-off point is very different (2.8 cm). So, this parameter must be taken with caution because it could depend on body mass index more than others.

To conclude with the ultrasound parameters, although some association was found between distance from hyoid bone and thyroid cartilage in the parasagittal plane with a cut-off point ≥ 1.23 cm and DL, it did not reach the statistical significance (*p* = 0.0589). We have also not found studies in which this parameter has been reflected, so it would be advisable to continue studying it.

CC ≥ 43 cm has been the only clinical predictor that showed significant association with DL (*p* = 0.0013) in our study. This result is like those found in different studies (21, 24, 25, 30, 31), which determines that as CC increases, the risk of DL progressively increases. Besides, a strong trend between male gender and DL (*p* = 0.0508) has been observed. This finding has also been described in other studies (19, 21, 25, 27, 32, 33). On the other hand, some authors (22–24, 34, 35) could not find a statistically significant correlation.

One of the strengths of this study is the creation of the risk score for the prediction of DL. The score presented a good discriminatory ability (AUC = 0.84). The categorized risk score with the cut-off point of 5 also showed good accuracy (AUC = 0.84). The likelihood of DL was 34 (95%CI, 4.15−278.42) times higher among those with risk score ≥ 5. Although the confidence interval for OR is wide, this may be explained due to the sample size. The cut-off point also showed a very good sensitivity of 91.67% and a specificity of 75.56% with PPV of 33.33% and NPV of 98.55%.

Other studies have also created associations of clinical and ultrasound parameters. The combination of different parameters improves their predictive capacity for DL instead of using only clinical or ultrasound parameters, as demonstrated in several studies (16, 21–23).

A strength of our study is the standardized ultrasound measurements performed in the sniffing position, which is the final position for endotracheal intubation by direct laryngoscopy. All clinical airway assessments and ultrasound measurements were performed by the same two investigators, improving the reliability of the data. Though, our study has several limitations. Firstly, patients with a known difficult airway were excluded, as they had to be intubated with equipment other than a conventional laryngoscope and Macintosh blade. In addition, patients who were intubated electively, either by teaching or by choice of the anaesthesiologist, using different technique than the one mentioned above were also excluded from the study. Only patients above 18 years were included, so the results of this study are not applicable in the pediatric setting. Finally, orotracheal intubation was performed by various experienced anaesthesiologists, so there might have been variability in the realization of the technique.

In conclusion, we can corroborate that the ultrasound distance between different laryngeal structures and the skin, and their combination with male gender are useful tools to predict difficult intubation. It should be noted that in almost all the published studies on ultrasound predictors of DL, the association between distance from skin to epiglottis and DL is strongly significant. Thus, we have created a risk score for DL which indicates the risk of DL 34 times higher for a score ≥ 5 points. Therefore, we consider that laryngeal ultrasonography is a useful tool for airway management. Nonetheless, new researches are necessary to confirm our results.

## Data availability statement

The original contributions presented in this study are included in this article/supplementary material, further inquiries can be directed to the corresponding author.

## Ethics statement

The studies involving humans were approved by the Ethical approval for this study (protocol N° 43.17 CEIHUB) was provided by the Clinical Ethic Committee of Basurto University Hospital, Bilbao, Spain, on 20th September 2017. The studies were conducted in accordance with the local legislation and institutional requirements. The participants provided their written informed consent to participate in this study. Written informed consent was obtained from the individual(s) for the publication of any potentially identifiable images or data included in this article.

## Author contributions

NL-C: Conceptualization, Investigation, Methodology, Validation, Visualization, Writing–original draft, Writing–review and editing. DL-L: Conceptualization, Supervision, Visualization, Writing–review and editing. PR-C: Supervision, Writing–review and editing. FS-M: Conceptualization, Project administration, Supervision, Validation, Writing–review and editing. AZ-LM: Supervision, Validation, Writing–review and editing. JH-H: Investigation, Writing–review and editing. EM-H: Supervision, Visualization, Writing–review and editing. RF-P: Validation, Writing–review and editing. AB-G: Writing–review and editing, Formal analysis. MF-V: Resources, Supervision, Validation, Visualization, Writing–review and editing.

## References

[1] Valero R Mayoral V Massó E López A Sabaté S Villalonga R Evaluación y manejo de la vía aérea difícil prevista y no prevista: Adopción de guías de práctica. *Rev Esp Anestesiol Reanim.* (2008) 55:563–70. 10.1016/S0034-9356(08)70653-4 19086724

[2] Nørskov A Rosenstock C Wetterslev J Lundstrøm L. Incidence of unanticipated difficult airway using an objective airway score versus a standard clinical airway assessment: the DIFFICAIR trial - trial protocol for a cluster randomized clinical trial. *Trials.* (2013) 14:347. 10.1186/1745-6215-14-347 24152537 PMC3842741

[3] Escobar J. Cuánto podemos predecir la vía aérea difícil? *Rev Chil Anest.* (2009) 38:84–90.

[4] Calder I. Identification of the difficult airway. *Anaesth Intens Care Med.* (2014) 15:355–7. 10.1016/j.mpaic.2014.04.011

[5] Merino M Marcos J García R Díez F España L Bermejo Evaluación de un protocolo de predicción de vía aérea difícil en la práctica habitual: estudio de concordancia. *Rev Esp Anestesiol Reanim.* (2010) 57:473–8. 10.1016/S0034-9356(10)70707-6 21033453

[6] Narula J Chandrashekhar Y Braunwald E. Time to add a fifth pillar to bedside physical examination. *JAMA Cardiol.* (2018) 3:346–50. 10.1001/jamacardio.2018.0001 29490335

[7] Diaz-Gomez J Mayo P Koening S. Point-of-care Ultrasonography. *N England J Med.* (2021) 17:1593–602. 10.1056/NEJMra1916062 34670045

[8] Kristensen M Teoh W Graumann O Laursen C. Ultrasonography for clinical decision-making and intervention in airway management: From the mouth to the lungs and pleurae. *Insights Imaging.* (2014) 5:253–79. 10.1007/s13244-014-0309-5 24519789 PMC3999368

[9] Sotoodehnia M Rafiemanesh H Mirfazaelian H Safaie A Baratloo A. Ultrasonography indicators for predicting difficult intubation: a systematic review and meta-analysis. *BMC Emerg Med.* (2021) 21:76. 10.1186/s12873-021-00472-w 34217221 PMC8254992

[10] Carsetti A Sorbello M Adrario E Donati A Falcetta S. Airway Ultrasound as Predictor of Difficult Direct Laryngoscopy: A Systematic Review and Meta-analysis. *Anesth Analg.* (2022) 134:740–50. 10.1213/ANE.0000000000005839 34914641 PMC8903216

[11] Gomes S Simões A Nunes A Pereira MV Teoh W Costa P Useful ultrasonographic parameters to predict difficult laryngoscopy and difficult tracheal intubation—a systematic review and meta-analysis. *Front Med.* (2021) 8:671658. 10.3389/fmed.2021.671658 34124099 PMC8193063

[12] Hanley A Mcneil J. The Meaning and Use of the area under a receiver operating characteristic (ROC) curve. *Diagn Radiol.* (1982) 143:29–36. 10.1148/radiology.143.1.7063747 7063747

[13] Hosmer D Lemeshow S Sturdivant R. *Applied logistic regression.* 3 edn. New York, NY: Wiley and Sons (2013). 10.1002/9781118548387

[14] Robin X Turck N Hainard A Tiberti N Lisacek F Sanchez J pROC: an open-source package for R and S+ to analyze and compare ROC curves. *BMC Bioinform.* (2011) 12:77. 10.1186/1471-2105-12-77 21414208 PMC3068975

[15] Bandos A Rockette H Gur DA. permutation test for comparing ROC curves in multireader studies: A multi-reader ROC, permutation test. *Acad Radiol.* (2006) 13:414–20. 10.1016/j.acra.2005.12.012 16554220

[16] Xu J Wang B Wang M Yao W Chen Y. The value of multiparameter combinations for predicting difficult airways by ultrasound. *BMC Anesthesiol.* (2022) 22:311. 10.1186/s12871-022-01840-0 36199026 PMC9533522

[17] Ni H Guan C He G Bao Y Shi D Zhu Y. Ultrasound measurement of laryngeal structures in the parasagittal plane for the prediction of difficult laryngoscopies in Chinese adults. *BMC Anesthesiol.* (2020) 20:134. 10.1186/s12871-020-01053-3 32487070 PMC7265219

[18] Lin J Bellinger R Shedd A Wolfshohl J Walker J Healy J Point of care ultrasound in airway evaluation and management: a comprehensive review. *Diagnostics.* (2023) 13:1541. 10.3390/diagnostics13091541 37174933 PMC10177245

[19] Fernandez-Vaquero M Charco-Mora P Garcia-Aroca M Greif R. Preoperative airway ultrasound assessment in the sniffing position: a prospective observational study. *Brazil J Anesthesiol.* (2022) 73:539–47. 10.1016/j.bjane.2022.07.003 35917848 PMC10533964

[20] Bhagavan S Nelamangala K. Accuracy of Preoperative Ultrasonographic Airway Assessment in Predicting Difficult Laryngoscopies in Adult Patients. *Cureus.* (2023) 15:1–7. 10.7759/cureus.35652 37009359 PMC10065457

[21] Pinto J Cordeiro L Pereira C Gama R Fernandes H Assunção J Predicting difficult laryngoscopy using ultrasound measurement of distance from skin to epiglottis. *J Crit Care.* (2016) 33:26–31. 10.1016/j.jcrc.2016.01.029 26948251

[22] Adhikari S Zeger W Schmier C Crum T Craven A Frrokaj I Pilot study to determine the utility of point of care ultrasound in the assessment of difficult laryngoscopy. *Acad Emerg Med.* (2011) 18:754–8. 10.1111/j.1553-2712.2011.01099.x 21707828

[23] Wu J Dong J Ding Y Zheng J. Role of anterior neck soft tissue quantifications by ultrasound in predicting difficult laryngoscopy. *Med Sci Monitor.* (2014) 20:2343–50. 10.12659/MSM.891037 25403231 PMC4247231

[24] Yadav N Rudingwa P Mishra S Pannerselvam S. Ultrasound measurement of anterior neck soft tissue and tongue thickness to predict difficult laryngoscopy - An observational analytical study. *Indian J Anaesth.* (2019) 63:629–34. 10.4103/ija.IJA_270_19 31462808 PMC6691631

[25] Martínez-García A Guerrero-Orriach J Pino-Gálvez M. Ultrasonography for predicting a difficult laryngoscopy. Getting closer. *J Clin Monit Comput.* (2020) 57:105–9. 10.1007/s10877-020-00467-1 31993893

[26] Parameswari A Govind M Vakamudi M. Correlation between preoperative ultrasonographic airway assessment and laryngoscopic view in adult patients: A prospective study. *J Anaesthesiol Clin Pharmacol.* (2018) 34:46–50. 29109635 10.4103/joacp.JOACP_166_17 PMC5672513

[27] Bouzid K Ketata S Zouche I Keskes M Fourati M Kammoun A Ultrasonography predicts difficult airway management: A prospective double blinded study. *Trends Anaesth Crit Care.* (2022) 46:18–24. 10.1016/j.tacc.2022.08.007

[28] Reddy P Punetha P Chalam K. Ultrasonography - A viable tool for airway assessment. *Indian J Anaesth.* (2016) 60:807–13. 10.4103/0019-5049.193660 27942053 PMC5125183

[29] Alessandri F Antenucci G Piervincenzi E Buonopane C Bellucci R Andreoli C Ultrasound as a new tool in the assessment of airway difficulties: An observational study. *Eur J Anaesthesiol.* (2019) 36:509–15. 10.1097/EJA.0000000000000989 31742568

[30] Ezri T Gewürtz G Sessler D Medalion B Szmuk P Hagberg C Prediction of difficult laryngoscopy in obese patients by ultrasound quantification of anterior neck soft tissue. *Anaesthesia.* (2003) 58:1111–4. 10.1046/j.1365-2044.2003.03412.x 14616599 PMC1283106

[31] Brodsky J Lemmens H Brock-Utne J Vierra M Saidman L. Morbid obesity and tracheal intubation. *Anesth Analg.* (2002) 94:732–6. 10.1097/00000539-200203000-00047 11867407

[32] Özdilek A Beyoglu C Erbabacan S Ekici B Altındaş F Vehid S. Correlation of neck circumference with difficult mask ventilation and difficult laryngoscopy in morbidly obese patients: an observational study. *Obes Surg.* (2018) 28:2860–7. 10.1007/s11695-018-3263-3 29687341

[33] Li X Lian Y Pan F Zhao H. Global research trends in prediction of difficult airways: A bibliometric and visualization study. *Medicine.* (2023) 102:1–6. 10.1097/MD.0000000000033776 37171310 PMC10174351

[34] Falcetta S Cavallo S Pelaia P Sorbello M. Ultrasound measurements as predictors of difficult direct laryngoscopy. *Trends Anaesth Critical Care.* (2017) 12:13–6. 10.1016/j.tacc.2017.01.008 29889671

[35] Komatsu R Sengupta P Wadhwa A Akça O Sessler D Ezri T Ultrasound quantification of anterior soft tissue thickness fails to predict difficult laryngoscopy in obese patients. *Anaesth Intens Care.* (2007) 35:32–7. 10.1177/0310057X0703500104 17323663

